# Tracking Neoantigens by Personalized Circulating Tumor DNA Sequencing during Checkpoint Blockade Immunotherapy in Non‐Small Cell Lung Cancer

**DOI:** 10.1002/advs.201903410

**Published:** 2020-03-26

**Authors:** Qingzhu Jia, Luting Chiu, Shuangxiu Wu, Jian Bai, Lina Peng, Linpeng Zheng, Rui Zang, Xueqin Li, Bibo Yuan, Yixing Gao, Dingyong Wu, Xiaohong Li, Lin Wu, Jianguo Sun, Ji He, Bruce W. S. Robinson, Bo Zhu

**Affiliations:** ^1^ Institute of Cancer Xinqiao Hospital The Army Medical University Xinqiao Main Street Chongqing 400037 China; ^2^ Chongqing Key Laboratory of Immunotherapy Xinqiao Main Street Chongqing 400037 China; ^3^ Berry Oncology Corporation NO. 4 Science Park Road, Changping District Beijing 102206 China; ^4^ GeneCast Biotechnology Co., Ltd. 35 Huayuan North Road, HealthWork Suite 901 Beijing 100191 China; ^5^ School of Medicine and Pharmacology University of Western Australia 35 Stirling Highway Perth Western Australia 6009 Australia; ^6^ National Centre for Asbestos Related Diseases GPO Box U1908 Perth Western Australia 6845 Australia

**Keywords:** ctDNA sequencing, immune checkpoint blockade, neoantigens, non‐small cell lung cancer, personalized medicine

## Abstract

The evolutionary dynamics of tumor‐associated neoantigens carry information about drug sensitivity and resistance to the immune checkpoint blockade (ICB). However, the spectrum of somatic mutations is highly heterogeneous among patients, making it difficult to track neoantigens by circulating tumor DNA (ctDNA) sequencing using “one size fits all” commercial gene panels. Thus, individually customized panels (ICPs) are needed to track neoantigen evolution comprehensively during ICB treatment. Dominant neoantigens are predicted from whole exome sequencing data for treatment‐naïve tumor tissues. Panels targeting predicted neoantigens are used for personalized ctDNA sequencing. Analyzing ten patients with non‐small cell lung cancer, ICPs are effective for tracking most predicted dominant neoantigens (80–100%) in serial peripheral blood samples, and to detect substantially more genes (18–30) than the capacity of current commercial gene panels. A more than 50% decrease in ctDNA concentration after eight weeks of ICB administration is associated with favorable progression‐free survival. Furthermore, at the individual level, the magnitude of the early ctDNA response is correlated with the subsequent change in tumor burden. The application of ICP‐based ctDNA sequencing is expected to improve the understanding of ICB‐driven tumor evolution and to provide personalized management strategies that optimize the clinical benefits of immunotherapies.

## Graphical abstract

Inter‐individual heterogeneity of mutation profiling is the primary obstacle in effective in vivo monitoring of mutation‐directed T‐cell killing using ctDNA sequencing. Herein, a customized sequencing strategy is developed that mainly targets predicted neoantigen‐coding mutations. This approach broadens the ctDNA sequencing coverage and is expected to improve personalized management strategies that optimize the clinical benefits from checkpoint immunotherapies.

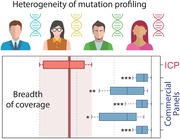

## 1 Introduction

The capacity of immune checkpoint blockade (ICB) to augment tumor‐specific T‐cell cytotoxicity has resulted in impressive improvements in clinical outcomes for patients with late‐stage solid tumors.^[^ ^1^ ^]^ Despite this key advance, a considerable portion of patients do not exhibit meaningful responses to ICB owing to drug resistance,^[^ ^2^ ^]^ immune‐related adverse events,^[^ ^3^ ^]^ or disease hyper‐progression.^[^ ^4^ ^]^ Effective and accurate monitoring of changes in tumor burden is vital for predicting responsiveness, making treatment decisions and understanding the dynamic evolution of tumor neoantigen profiles that occur during therapy.

The level of circulating tumor DNA (ctDNA), a surrogate of tumor burden, can be used to estimate clinical responses in patients receiving anti‐tumor treatment.^[^ ^5^, ^6^, ^7^, ^8^ ^]^ In a longitudinal assessment, the baseline concentration and early mutational dynamics of ctDNA were correlated with radiological and survival outcomes in response to ICB treatment in multiple solid tumor types.^[^ ^9^, ^10^, ^11^, ^12^, ^13^, ^14^, ^15^ ^]^ Typically, commercially available panels for ctDNA mutation profiling cover a limited set of genes, such as known oncogenes, tumor suppressor genes, immune‐related genes, and other targets of actionability and/or research interest. However, there is mounting evidence that mutated neoantigens are the main targets of immune attack during ICB immunotherapy. Therefore, dynamic tracking of ctDNA mutations resulting in neoantigens would be of higher value than those of irrelevance. Given that T‐cell‐recognizable neoantigens are largely tumor‐specific owing to the highly patient‐dependent genetic background, personal HLA genotype,^[^ ^16^ ^]^ history of environmental exposure,^[^ ^17^ ^]^ and other factors, a predefined generic panel would face challenges in sufficiently cover these dynamics. While panel per se one might consider a “totality” approach such as whole exome sequencing (WES) or even whole genome sequencing (WGS), in reality, deep sequencing (which is required for effective mutation detection from ctDNA) of WES and WGS would become cost prohibitive. Additionally, requirement on input DNA quantity for WES or WGS makes their clinical application to ctDNA hardly practicable. Understandably, this challenge could be addressed by an individually customized panel (ICP) with a reasonable DNA input requirement and at a manageable cost, that at the same time ensures a tailored coverage of patient‐specific dominant neoantigens, with the dominant neoantigens in silico‐predicted from a one‐time sequencing of matched tumor tissue using WES.

With this understanding, in this study, we designed primary tumor‐guided personalized neoantigen panels for ctDNA sequencing to monitor the clinical response to first‐line ICB treatment for patients with advanced non‐small cell lung cancer (NSCLC). We demonstrated that this strategy enables the monitoring of early response, prediction of the treatment efficacy and disease relapse, and tracking of neoantigen evolution during the course of ICB treatment.

## 2 Result

### 2.1 Individually Customized Panels Enable Tailored Coverage of Target Genes in ctDNA Sequencing

**Figure 1:**
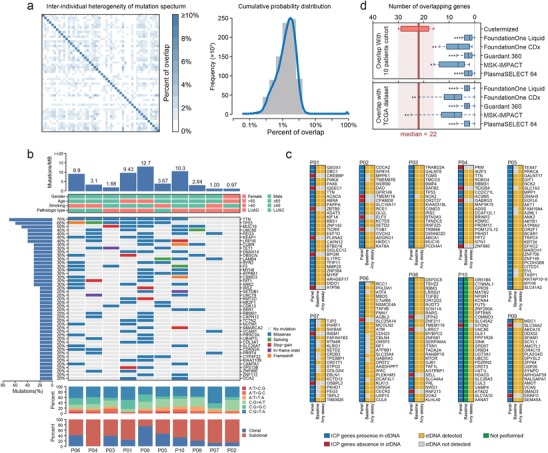
Somatic mutation profiles of pre‐treatment tissues and matched blood samples for ten patients with non‐small cell lung cancer (NSCLC). a) Left, heatmap showing inter‐individual overlap in the mutation spectrum for cases in the NSCLC dataset of TCGA. The percentage of overlap was calculated as the ratio between the numbers of intersected and union genes. Color bar, percentage of overlap. For clear visualization, 50 patients randomly selected from 1059 cases are shown. Right, histogram showing the distribution of percentages of overlap for all patients with NSCLC in TCGA. Blue line, fitting curve. b) Mutant genes detected in more than one patient in the cohort are illustrated. Total mutation burden (per MB) and clinical information are annotated in the upper two panels. Gene names are labeled on the right. The substitution spectrum and composition of clonal/subclonal mutations for all detected mutations are shown in the lower two panels. c) The heatmap shows an overview of individually customized panels (ICPs) and follow‐up ctDNA sequencing for each patient. The left column represents all genes in the ICP; the middle column summarizes ctDNA sequencing results for baseline samples; the right column indicates genes for which ctDNA could be detected in the following assays at least once. Blue square, genes detected over the duration of the treatment; red square, genes never detected in any following ctDNA sequencings; yellow square, ctDNA detected; grey square, ctDNA not detected; green square, sequencing not performed. d) Number of overlapping genes between the whole exome sequencing (WES)‐based mutation spectrum and panel gene lists. Upper panel, mutation spectrum obtained from the 10 patients; lower panel, mutation spectrum obtained from the NSCLC dataset of TCGA. Red box, number of genes detected by ICP‐based sequencing during the course of treatment; blue box, virtual validation of commercial panels, number of genes shared between the commercial panel gene list and patient mutation spectra. The boxplot shows the median value with ranges. *p‐*values are based on Kruskal–Wallis tests; for comparisons between the red box and each labeled box: *p < 0.05, **p < 0.01, ***p < 0.001. The red vertical line and red shadow indicate the median and range for the red box.

It is a general perception that the spectrum of somatic mutations in tumors might vary considerably among individuals. Part of our study aimed to gain a more quantified understanding of such variability. We first investigated how many mutated genes from two patients overlapped with each other. We measured the overlap as the ratio in percentage of the number of commonly mutated genes between two patients over the total number of mutated genes across both patients. Based on 1059 patients with NSCLC in The Cancer Genome Atlas (TCGA) dataset, **Figure** **1**a depicts in detail the percentage of overlap between any patient pair out of a random cohort of 50 patients (Figure 1a, left panel) and summarizes the distribution of the overlap for all 1059 patients (Figure 1a, right panel). The median percentage of overlap between any two patients was as low as 1.21% (lower quantile, 0.61%; upper quantile, 1.88%), which has confirmed the substantial heterogeneity in the spectrum of non‐synonymous somatic mutations in the TCGA NSCLC cohort. When focusing on different pathologic types, we also identified consistent highly inter‐patients heterogeneity for both lung adenocarcinoma (LUAD) and lung squamous cell cancer (LUSC) (Figure S1, Supporting Information. 1.34% for LUAD and 2.11% for LUSC). For patients enrolled in our cohort (Summarized in Table S1, Supporting Information), genomic profiling based on WES revealed a total of 2119 mutated genes, ranging from 38 to 496 per patient (Figure 1b and Table S2, Supporting Information). TTN, TP53, and MUC16 were found to be mutated in more than half of the cohort, which is consistent with the reported mutation prevalence of these genes in the population (Figure 1b). However, only 45 and 19 mutated genes were shared between two patients and more than three patients, accounting for 2.12% and 0.90% of all mutated genes, respectively, further confirming the same level of remarkable differences in the mutation spectra (Table S3, Supporting Information). Findings on these cohorts strongly suggest that a personalized detection strategy in form of an ICP is required to effectively cover the mutations of each individual patient.

In order to construct an ICP in our study, putative neoantigens were first predicted in silico based on WES data using previously established approaches.^[^ ^18^, ^19^ ^]^ A median of 28.5 (ranging from 2 to 189) dominant neoantigens was identified per patient (Table S4, Supporting Information). The top 20–30 neoantigen‐coding genes with the highest affinity scores were included into the ICP panels following to the detailed procedure in Figure S2, Supporting Information. This ICP strategy accounted for a median of 82.67% of all predicted neoantigens (ranging from 15.87% to 100%) per patient. We then performed on‐treatment monitoring of the individual based on ctDNA sequencing using their corresponding ICP. The concordance between the ICP‐detected neoantigens in ctDNA and the WES‐detected ones in tumor tissue is summarized in grid heatmaps (Figure 1c). Overall, a median of 80% of mutations (ranging from 35% to 95%) originally incorporated into the ICPs was successfully detected in circulation at the baseline sampling point, whereas during the entire treatment period, a median of 90% of mutations incorporated into the ICP (ranging from 80% to 100%) was detected for at least once, demonstrating the reliability of ICP‐based ctDNA sequencing for the tailored tracking of previously predicted neoantigens in patients with NSCLC (Figure 1c and Figure S3, Supporting Information).

Through in silico simulation, the effectiveness of ICP panels in covering patient‐specific neoantigens was compared against those of five commercially available panels including Foundation Liquid, FoundationOne CDx, Guardant 360, MSK‐IMPACT, and PlasmaSELECT 64 (two genes in FoundationOne CDx were inapplicable and therefore excluded, see details in Table S5, Supporting Information). The numbers of overlapping genes between each commercial panel and the WES‐derived mutation spectrum for patients in our cohort were calculated. The number of overlapping genes was significantly lower for all five commercially available panels than for the ICP (Figure 1d, upper panel). To further eliminate potential bias due to ethnic differences in the mutation spectrum (i.e., differences between Asian and Caucasian/Latino populations with NSCLC), genomic profiling data for NSCLC cases from TCGA were used for validation using these commercially available panels. Still, a substantially smaller percentage of overlapping genes was covered by either panel (Figure 1d, lower panel). These comparisons proved the superiority of ICP for personalized neoantigen monitoring.

### 2.2 Association Between Clinical Parameters and Neoantigens in ctDNA

Numerous studies have demonstrated that baseline ctDNA can be used as an approximate indicator of tumor burden in patients with metastases for a wide range of tumor types.^[^ ^20^, ^21^, ^22^ ^]^ In our cohort, tumor burden also correlated with the ctDNA load measured based on either the variant allele frequencies of the individually detected mutations by ICP (Figure S4, Supporting Information, left panel, *R* = 0.479, ***p = 1.39 × 10^−14^) or their mean values (Figure S4, Supporting Information, right panel, *R* = 0.464, *p* = 0.0993) for each patient. Furthermore, ctDNA levels showed a greater decline in patients who experienced an objective response to ICB at eight weeks after the first infusion than in patients who showed no response (Figure S5, Supporting Information, *p = 0.0238). We next investigated whether the early decrease in peripheral ctDNA could predict prolonged survival in response to immunotherapy, as reported in ICB‐treated cohorts with relatively large cohort sizes.^[^ ^12^, ^14^, ^23^ ^]^ Surprisingly, even with a small sample size of nine patients (P10 was excluded) with baseline blood samples in our cohort, the substantial early decrease in ctDNA abundance (≥50% decrease at eight weeks) predicted prolonged progression‐free survival after ICB administration (Figure S6, Supporting Information, **p = 0.003 and HR = 6.005 [0.879–41.010]). These observations strongly support the use of ICP‐based ctDNA sequencing to identify patients likely to have favorable outcomes following ICB treatment.

### 2.3 Concordance between Measurable Tumor Burden and Serial Neoantigens in ctDNA

**Figure 2:**
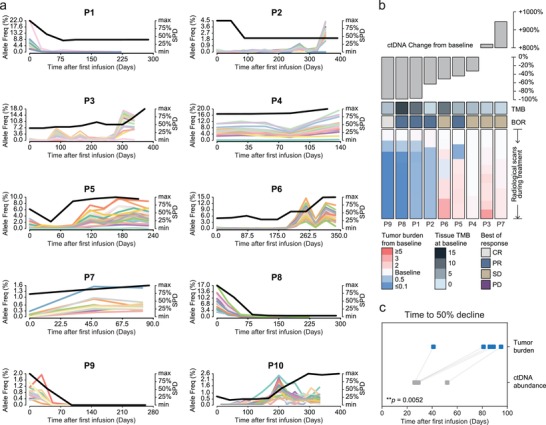
Tumor burden with respect to ctDNA changes during the course of follow‐up. a) Sequential neoantigen ctDNA‐assessment and tumor burden during treatment. The dynamic changes in the ctDNA allele frequencies are presented as lines of different colors. Tumor burden was quantified as the sum of products of perpendicular diameters (SPDs), and illustrated as black lines for each patient. SPDs were subjected to linear scaling. b) Correlation between the magnitude of the ctDNA decline and clinical outcomes after immune checkpoint blockade (ICB) administration. Grey bar plot in upper panel, fold‐change in mean ctDNA minor allele frequency (MAF) from the baseline to 12 weeks after first cycle of administration. Lower panel, heatmap showing the trend in radiological imaging‐based SPD‐measured tumor burden from the baseline evaluation to the last radiological follow‐up. Each lane represented the tumor burden for one patients serially. Each color square within the lane represented the tumor burden in each surveillance scan. The baseline tissue tumor mutational burden (TMB) and best of response (BOR) for each patient are annotated above the heatmap. The color gradient indicates the change in tumor burden. Patients are ordered according to the decline in ctDNA at 12 weeks. c) Time to tumor burden versus ctDNA decline among patients with radiological confirmation of >50% SPD decline. Statistics are based on two‐tailed paired Student's *t*‐tests.

In addition to its use as a predictive indicator at the population level, we investigated whether the ICP‐based assessment of neoantigen level changes could be indicative of quantitative changes in tumor burden at the individual level. The radiological tumor burden response (determined by the sum of products of perpendicular diameters (SPDs)) and longitudinal ctDNA kinetics was evaluated, with the results shown in **Figure** **2**a (response evaluation criteria in solid tumor (RECIST)‐measured tumor burden showed a consistent association with ctDNA change in Figure S7, Supporting Information). Tumor burden was quantitatively compared to ctDNA levels at the time point nearest to that of the surveillance scans (Figure S8, Supporting Information). Based on the SPD‐measured tumor burden, changes in ctDNA after ICB administration satisfactorily reflected the degree of radiological tumor burden in eight of ten patients, with *R* = 0.509–0.999, strongly supporting the potential use of ICP to monitor the dynamics of tumor burden through ctDNA sequencing (Figure S8, Supporting Information). Outliers including patient 02 and patient 10 are further discussed in the following section.

The magnitude of ctDNA decline varied substantially among individuals when measured by ICP (Figure 2b upper panel). When patients were ordered according to the magnitude of ctDNA decline, responders with a persistent clinical benefit were substantially over‐represented among patients with greater declines at the ctDNA level during ICB therapies, yet were under‐represented among patients who showed relatively less changes (Figure 2b). Similarly, when ordered according to the magnitude of the change in tumor burden, responders were relatively over‐represented among patients with greater reductions in ctDNA (Figure S9, Supporting Information). Furthermore, the average time to a 50% decline in tumor burden was 32.4 days if determined by the ICP‐measured ctDNA level but was 78.4 days if determined by radiological evaluation (Figure 2c). These interesting observations suggest that ICP‐based ctDNA sequencing could become an effective clinical utility to identify responsiveness and to indicate the survival benefit of ICB, and furthermore, it might serve as a more sensitive surveillance platform when compared with radiological scanning, as it could predict prognosis at a significantly earlier timepoint.

### 2.4 Case Presentation for Outliers

**Figure 3:**
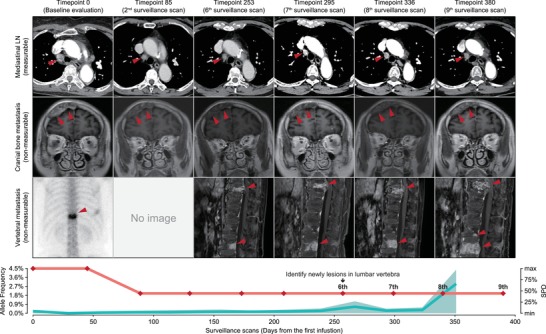
Radiological and serological follow‐ups for P2 with unmeasurable lesions. Upper panels, surveillance CT, MRI scans (with contrast), or ECT showing the change in tumor burden over time in the mediastinal lymph node (LN), cranial bone, and lumbar vertebrae. Red arrowheads indicate the lesion site. Lower panel, clinical course. Red lines, SPD; green line with shadow, mean ctDNA MAF with the range for all detected mutations in ctDNA.

Patient 02 was a 68‐year‐old female diagnosed with lung adenocarcinoma and T2N1M1c stage IV disease. She was enrolled in the NEPTUNE trial (NCT02542293) that tested the efficacy of tremelimumab/durvalumab combinational immunotherapy and achieved a partial response after four cycles of administration. Baseline emission computed tomography revealed an unmeasurable metastatic lesion in the 12th thoracic vertebra (**Figure** **3**, upper panel) according to the RECIST 1.1 criteria. Following marked tumor shrinkage in the mediastinal metastatic lymph node, the ctDNA level gradually increased from 239 days after the first infusion, reaching a 14‐fold elevated level when compared with the baseline level at 351 days (Figure 3, lower panel). The re‐elevation of cancer embryonic antigen (CEA) levels that was observed also strongly suggested disease progression during treatment (Figure S10, Supporting Information). To assess symptomatic vertebral metastases, magnetic resonance imaging with contrast was performed at nine months after the baseline evaluation and this revealed a newly unmeasurable lesion in the 5th lumber vertebra. Although the lesion could not be considered measurable and was not included in the SPD‐assessed tumor burden, the increased ctDNA levels that were observed were accompanied by the gradual progression of bone metastases.

Patient 10 was a 65‐year‐old male without a history of smoking. He was diagnosed with adenocarcinoma and also enrolled in the NEPTUNE trial. Because the researchers judged that the subject would still benefit from the drugs, he continued to receive ICB‐agents after the radiological progression of disease. Sequencing analysis showed that he experienced rapid progression of tumor burden and a paradoxical decline in ctDNA load (Figure 2a, panel for P10). We have not determined the exact reason for this decline, but the growth of treatment‐resistant clones is one potential explanation. Interestingly, the ctDNA of all ICP‐covered neoantigens disappeared suggesting potential outgrowth of a different clone that did not bear these neoantigens, rather than a subclone, which was not sufficiently represented in the original biopsy.

## 3 Discussion

A number of sophisticated approaches have been developed with an optimized panel design, such as cancer personalized profiling by deep sequencing (CAPP‐Seq)^[^ ^24^ ^]^ and integrated digital error suppression^[^ ^25^ ^]^ CAPP‐Seq, to increase the sensitivity of mutation detection in circulating tumor DNA. However, such approaches were typically based on a fixed set, generic targeted panel design, with relatively less attention paid to the flexibility of mutation coverage, risking the potential exclusion of clinically meaningful mutations. Alternatively, personalized targeted sequencing, in which the sequencing amplicons are designated based on genomic alterations identified from diagnostic tumor tissues, is being studied as an avenue to improve the breadth of coverage in ctDNA monitoring.^[^ ^26^, ^27^, ^28^ ^]^ In this study, we streamlined a modified protocol for ctDNA sequencing to cover dominant in silico‐predicted neoantigens and track the dynamic evolution of these neoantigen‐coding mutations in response to ICB immunotherapies. ICP‐based neoantigens were detected in all nine patients with available baseline blood samples. The average number of detected mutations of 22 per patient in our assay is an improvement over previous clinical approaches with commercially available panels (Figure 1c and Figure S3, Supporting Information). In silico validation results have also confirmed the advantage of ICP‐based sequencing with respect to the breadth of coverage in comparison with various commercially available panels (Figure 1d).

We further applied the ICP‐based ctDNA sequencing for real‐time on‐treatment biomarker monitoring and achieved improved tracking of neoantigen evolution with the following evidence: 1) Early changes in ctDNA predicted tumor load dynamics during treatment (Figure 2a). In particular, for patients who achieved a 50% decline in tumor burden, the ctDNA response was detected prior to radiologically confirmed disease control (Figure 2c). 2) The magnitude of ctDNA load decline was positively associated with tumor shrinkage measured by radiological scans (Figure 2b). Although at the cohort level, patients with substantial ctDNA responses (for which the definition varies in different publications) usually showed better tumor control with immunotherapy, our studies are among the first to witness this correlation more precisely to an individual level. 3) An unaccountable elevation in ctDNA load might suggest progressive disease, particularly caution the new lesion that is outside the field of routine surveillance scan (Patient 02). 4) ICP is potentially applicable for discriminating relapsed patients owning to neoantigen loss (Patient 10). Lastly, it is worthwhile to mention that despite lacking a representative case in our present cohort, theoretically, ICP‐based detection would be capable of discriminating true progression from pseudo‐progression for enlarged lesions with a stable ctDNA load. Although on a flip side, owing to the nature of ICP design and the heterogeneity of tumor environment, ICP would lack the predictive value in discriminating pseudo‐progression from true progression.

In addition to these prospects, this neoantigen‐coding mutation‐based ICP also helps identify the underlying mechanism of immune editing during immunotherapy. We found that the general trend of ctDNA kinetics for each involved gene is almost parallel, strongly suggesting a broad spectrum of T‐cell‐mediated attack associated with ICB immunotherapies, instead of targeting a few “key” epitopes in the anti‐tumor response. However, selective clonal evolution and subsequent acquired resistance during treatment have long been a concern, especially in the era of targeted therapies.^[^ ^29^, ^30^, ^31^ ^]^ Immunotherapies can result in evolution of the neoantigenic landscape, consistent with the theory of immunoediting.^[^ ^32^ ^]^ An analysis of matched pre‐ and post‐treatment tumor samples by Valsamo and colleagues^[^ ^33^ ^]^ found that 7–18 putative neoantigens are eliminated in immunotherapy‐resistant cases, providing the first evidence that neoantigen evolution might be involved in acquired resistance to immunotherapy. Interestingly, in our cohort, except for that observed for one patient (Patient 10), initial neoantigen‐coding mutations could still be detected at the stage of relapse in nine patients, suggesting that a sufficiently large‐scale of neoantigen loss might be attributable to just a small portion of relapsed patients after ICB treatment. The difference in observations between Valsamo's and our study could be due to the spatial heterogenous^[^ ^34^, ^35^ ^]^ mutation spectrum of tumors. In three of four patients in Valsamo's cohort, mutations were estimated based on matched tumor tissues sampled from proximate but different anatomical sites, and this discrepancy could be overcome by circulating assays such as ctDNA sequencing. Therefore, for patients without neoantigen loss, we propose that strategies that support or re‐provoke the activation and expansion of neoantigen‐specific T‐cells might help to overcome resistance after ICB treatment. Besides checkpoint blockade immunotherapies, neoantigen‐based personal vaccination has long been envisioned as an effective stimulus to provoke tumor‐specific T cell responses.^[^ ^36^ ^]^ However, despite being facilitated by the progress of high‐throughput sequencing and bioinformatic tools, the clinical effectiveness of extensively past work was far from ideal. We believe that personalized ctDNA sequencing is an excellent tool to monitor the clearance of neoantigen‐expressing tumor cells and to gain mechanistic insight into resistance to neoantigen‐based cancer vaccines.

One limitation of ICP‐based neoantigen tracking during immunotherapy probably lies in the pipeline for the in silico prediction of neoantigens. Current algorithms predict the peptide‐MHC binding affinity but cannot accurately predict proteasomal processing, post‐proteasomal trimming, and whether sufficient peptide‐MHC complexes reach the cell surface. Therefore, all neoantigen prediction strategies only provide a putative list with the greatest possibility to cover most bona fide tumor‐expressing neoantigens. Furthermore, the lead time for the clinical application of ICP, including WES, neoantigen prediction, and the synthesis of patient‐specific sequencing probes, constrains our ability to make real‐time neoantigen tracking observations in the first two cycles of ICB administration. Presently, this study was limited in its cohort size and was performed for feasibility assessment purposes, partly due to the technical demands of the approach. The clinical significance of ICP‐based ctDNA sequencing needs to be validated with a larger cohort, with a prospective trial design and long‐term surveillance scans, after which, ICP‐based ctDNA sequencing could potentially improve our understanding of neoantigenic evolution and minimize unnecessary ICB‐mediated immunological toxicity, ultimately maximizing the clinical benefits of checkpoint blockade immunotherapies.

## 4 Conclusion

ICP‐based ctDNA sequencing provides a superior coverage to longitudinally track predicted dominant neoantigens. The clinical application of such an approach will warrant personalized management strategies that in turn improve the clinical benefits of immunotherapies and enhance our understanding of ICB‐driven tumor editing and neoantigenic profile evolution.

## 5 Experimental Section

##### Patients and Sampling

Ten patients diagnosed with stage IIIB/IV non‐small cell lung cancer (NSCLC) were treated with durvalumab monotherapy or combination therapy with tremelimumab/durvalumab (clinical information summarized in Table S1, Supporting Information). All patients were confirmed to lack an *EGFR* mutation or *ALK‐EML4* fusion at Chongqing Xinqiao Hospital. Ten formalin‐fixed, paraffin‐embedded (FFPE) tumor tissues and matched peripheral blood samples were obtained prior to the first infusion of ICB. During the course of treatment, 97 time‐course blood samples (including baseline samples) were collected for ctDNA sequencing. Clinical responses were evaluated by a senior physician based on RECIST 1.1 criteria every six weeks (two cycles of ICB administration). Tumor burden was measured by the sum of the longest diameter of evaluable lesions according to RECIST 1.1 criteria (designated as RECIST‐measured tumor burden), or by the sum of the product of perpendicular diameter of evaluable lesions (designated as SPD‐measured tumor burden). The study protocol was approved by the institution review board of Xinqiao Hospital, Army Medical University. Written informed consent for sample acquisition was obtained from all patients. All data were deidentified.

##### Tissue and Plasma DNA Isolation and Purification

Genomic DNA (gDNA) was extracted from the FFPE samples using the GeneRead DNA FFPE Kit (Qiagen, Germantown, MD, USA) and from peripheral blood mononuclear cells using the DNA Blood Midi/Mini kit (Qiagen) according to the manufacturer's instructions. Plasma cell‐free DNA (cfDNA) was isolated using the MagMAX Cell‐Free DNA Isolation Kit (Thermo, Waltham, MA, USA) according to the manufacturer's protocol. The quality of purified DNA was assayed by gel electrophoresis and quantified with a Qubit 4.0 Fluorometer (Life Technologies, Carlsbad, CA, USA).

##### Library Construction and ctDNA and Whole Exome Sequencing

Purified gDNA was first fragmented into DNA pieces of approximately 300 bp using an enzymatic method (5 × WGS Fragmentation Mix; Qiagen). After end‐repairing and A tailing, T‐adaptors were ligated on both ends, followed by PCR amplification to obtain a pre‐library. The final sequencing libraries were prepared using the 96 rxn xGen Exome Research Panel v1.0 (Integrated DNA Technology, Coralville, IA, USA) according to the manufacturer's protocol. For the targeted sequencing of cfDNA, the pre‐libraries were prepared according to the method previously described.^[^ ^37^ ^]^ In‐house panels were designed to capture cfDNA fragments to generate sequencing libraries. The sequencing libraries were evaluated using the NovaSeq 6000 platform (Illumina, San Diego, CA, USA) in 150PE mode.

##### Bioinformatics Analysis of Whole Exome Sequencing Results

The raw sequencing reads were subjected to quality control by trimming adaptor sequences and removing poly‐N sequences (>10%) and low‐quality reads (<Q20) preprocessed using FASTP.^[^ ^38^ ^]^ The FASTQ files were aligned to the human reference genome (hg19/GRCh37) using Burrows–Wheeler Aligner (BWA, v0.7.15).^[^ ^18^ ^]^ Picard (2.12.1) (https://picard.sourceforge.net/) was used to process PCR duplicates for mapped BAM files. GATK (the Genome Analysis Toolkit 3.8)^[^ ^39^ ^]^ was used for local realignment and base quality recalibration was employed to compute sequencing coverage and depth. Single nucleotide variants (SNVs) and small insertions and deletions were identified using GATK MuTect2. Mutations in the ENCODE Data Analysis Consortium blacklist were removed.^[^ ^40^ ^]^

Variants were annotated using ANNOVAR^[^ ^41^ ^]^ based on multiple databases, including HGVS variant description and population frequency databases (1000G, ExAC, and dbSNP), disease or phenotype databases (OMIM, COSMIC, and ClinVar), and variant functional in silico prediction tools (PolyPhen‐2 and SIFT). After annotation, SNVs annotated as genomicSuperDups with a variant allele frequency (VAF) < 0.2 or PopFreqMax > 0.05 were excluded and nonsynonymous SNVs with a VAF > 3% or with a VAF > 1% in cancer hotspots collected from patient databases were retained for further analyses.

For each mutation, the proportion of mutated reads (variant allele fraction, VAF), the proportion of tumor cells harboring the mutation (cancer cell fraction, CCF), and the clonality were calculated according to the methods described by Letouzé et al.^[^ ^40^ ^]^ We applied a binomial test to compute the 95% confidence interval (CI) of VAF, which was used to calculate the 95% CI of CCF thereafter. A mutation was regarded as subclonal if the upper boundary of the 95% CI of CCF was <1, and clonal otherwise. Tumor mutation burden was defined as the total number of non‐synonymous SNVs per megabase of coding sequence in a tumor genome based on WES.

##### Neoantigen Prediction and Individual Personal Mutation Probe Design

Dominant neoantigens were predicted using two computational tools, OptiType^[^ ^42^ ^]^ to infer the individual HLA type and pVAC‐Seq^[^ ^19^ ^]^ to predict the binding affinity of non‐silent mutant peptides. To reduce redundancy and select neoepitopes predicted to exhibit strong specific HLA‐peptide binding, 500 nm MHC binding affinity was applied as a filtering criterion. The predicted neoantigens were sorted by their binding score. For individual patients with more than 20 predicted neoantigens, the neoantigens with the top 30 binding scores were selected to design DNA probes to assay their presence and abundance in plasma during ICB treatment. For individual patients with fewer than 20 predicted neoantigens, all predicted neoantigens were selected, supplemented with mutations with the highest VAFs to obtain a total of 20 loci.

##### Bioinformatics Analysis of cfDNA Mutations

FASTP^[^ ^38^ ^]^ was used to trim adapters and to remove low‐quality sequences to obtain clean reads. The clean reads were aligned against the Ensemble GRCh37/hg19 reference genome using BWA.^[^ ^18^ ^]^ PCR duplicates were processed using gencore, and consensus reads were generated. SAMtools^[^ ^43^ ^]^ was applied for the detection of SNVs, insertions, and deletions. HGVS variants were annotated using ANNOVAR.^[^ ^41^ ^]^

##### Statistical Analysis

Statistical analyses and data visualization were conducted using R/Bioconductor packages. The Kaplan–Meier method was used for the survival analysis, median values were compared using log rank tests, and hazard ratios were obtained from the Cox proportional hazards model. Overlapping genes between ICP, WES, and commercially available panels were evaluated by Kruskal–Wallis tests. Dunn's test was used for post‐hoc analyses, and *p*‐values were adjusted based on the Benjamini–Hochberg correction.

## Conflict of Interest

The authors declare no conflict of interest.

## Supporting information

Supporting Information

Supporting TableS1

Supporting TableS2

Supporting TableS3

Supporting TableS4

Supporting TableS5

## References

[1] P. Darvin , S. M. Toor , V. Sasidharan Nair , E. Elkord , Exp. Mol. Med. 2018, 50, 1. 10.1038/s12276-018-0191-1 PMC6292890 30546008

[2] P. Sharma , S. Hu‐Lieskovan , J. A. Wargo , A. Ribas , Cell 2017, 168, 707. 28187290 10.1016/j.cell.2017.01.017 PMC5391692

[3] C. F. Friedman , T. A. Proverbs‐Singh , M. A. Postow , JAMA Oncology 2016, 2, 1346. 27367787 10.1001/jamaoncol.2016.1051

[4] S. Kato , A. Goodman , V. Walavalkar , D. A. Barkauskas , A. Sharabi , R. Kurzrock , Clin. Cancer Res. 2017, 23, 4242. 28351930 10.1158/1078-0432.CCR-16-3133 PMC5647162

[5] X. Yi , J. Ma , Y. Guan , R. Chen , L. Yang , X. Xia , Int. J. Cancer. 2017, 140, 2642. 28124376 10.1002/ijc.30620 PMC5434851

[6] M. R. Ossandon , L. Agrawal , E. J. Bernhard , B. A. Conley , S. M. Dey , R. L. Divi , P. Guan , T. G. Lively , T. C. McKee , B. S. Sorg , J. V. Tricoli , JNCI, J. Natl. Cancer Inst. 2018, 110, 929. 29931312 10.1093/jnci/djy105 PMC6136923

[7] K. Pantel , C. Alix‐Panabieres , Nat. Rev. Clin. Oncol. 2019, 16, 409. 30796368 10.1038/s41571-019-0187-3

[8] L. Cabel , C. Proudhon , E. Romano , N. Girard , O. Lantz , M. H. Stern , J. Y. Pierga , F. C. Bidard , Nat. Rev. Clin. Oncol. 2018, 15, 639. 30050094 10.1038/s41571-018-0074-3

[9] E. J. Lipson , V. E. Velculescu , T. S. Pritchard , M. Sausen , D. M. Pardoll , S. L. Topalian , L. A. Diaz Jr. , J. ImmunoTher. Cancer 2014, 2, 42. 25516806 10.1186/s40425-014-0042-0 PMC4267741

[10] J. H. Lee , G. V. Long , S. Boyd , S. Lo , A. M. Menzies , V. Tembe , A. Guminski , V. Jakrot , R. A. Scolyer , G. J. Mann , R. F. Kefford , M. S. Carlino , H. Rizos , Ann. Oncol. 2017, 28, 1130. 28327969 10.1093/annonc/mdx026

[11] L. Cabel , F. Riva , V. Servois , A. Livartowski , C. Daniel , A. Rampanou , O. Lantz , E. Romano , M. Milder , B. Buecher , S. Piperno‐Neumann , V. Bernard , S. Baulande , I. Bieche , J. Y. Pierga , C. Proudhon , F. C. Bidard , Ann. Oncol. 2017, 28, 1996. 28459943 10.1093/annonc/mdx212

[12] J. H. Lee , G. V. Long , A. M. Menzies , S. Lo , A. Guminski , K. Whitbourne , M. Peranec , R. Scolyer , R. F. Kefford , H. Rizos , M. S. Carlino , JAMA Oncology. 2018, 4, 717. 29423503 10.1001/jamaoncol.2017.5332 PMC5885201

[13] G. J. Weiss , J. Beck , D. P. Braun , K. Bornemann‐Kolatzki , H. Barilla , R. Cubello , W. Quan Jr. , A. Sangal , V. Khemka , J. Waypa , W. M. Mitchell , H. Urnovitz , E. Schutz , Clin. Cancer Res. 2017, 23, 5074. 28320758 10.1158/1078-0432.CCR-17-0231

[14] S. B. Goldberg , A. Narayan , A. J. Kole , R. H. Decker , J. Teysir , N. J. Carriero , A. Lee , R. Nemati , S. K. Nath , S. M. Mane , Y. Deng , N. Sukumar , D. Zelterman , D. J. Boffa , K. Politi , S. N. Gettinger , L. D. Wilson , R. S. Herbst , A. A. Patel , Clin. Cancer Res. 2018, 24, 1872. 29330207 10.1158/1078-0432.CCR-17-1341 PMC5899677

[15] R. Raja , M. Kuziora , P. Z. Brohawn , B. W. Higgs , A. Gupta , P. A. Dennis , K. Ranade , Clin. Cancer Res. 2018, 24, 6212. 30093454 10.1158/1078-0432.CCR-18-0386

[16] M. Luksza , N. Riaz , V. Makarov , V. P. Balachandran , M. D. Hellmann , A. Solovyov , N. A. Rizvi , T. Merghoub , A. J. Levine , T. A. Chan , J. D. Wolchok , B. D. Greenbaum , Nature 2017, 551, 517. 29132144 10.1038/nature24473 PMC6137806

[17] Z. R. Chalmers , C. F. Connelly , D. Fabrizio , L. Gay , S. M. Ali , R. Ennis , A. Schrock , B. Campbell , A. Shlien , J. Chmielecki , F. Huang , Y. He , J. Sun , U. Tabori , M. Kennedy , D. S. Lieber , S. Roels , J. White , G. A. Otto , J. S. Ross , L. Garraway , V. A. Miller , P. J. Stephens , G. M. Frampton , Genome Med. 2017, 9, 34. 28420421 10.1186/s13073-017-0424-2 PMC5395719

[18] H. Li , R. Durbin , Bioinformatics 2009, 25, 1754. 19451168 10.1093/bioinformatics/btp324 PMC2705234

[19] J. Hundal , B. M. Carreno , A. A. Petti , G. P. Linette , O. L. Griffith , E. R. Mardis , M. Griffith , Genome Medicine. 2016, 8, 11. 26825632 10.1186/s13073-016-0264-5 PMC4733280

[20] C. Bettegowda , M. Sausen , R. J. Leary , I. Kinde , Y. Wang , N. Agrawal , B. R. Bartlett , H. Wang , B. Luber , R. M. Alani , E. S. Antonarakis , N. S. Azad , A. Bardelli , H. Brem , J. L. Cameron , C. C. Lee , L. A. Fecher , G. L. Gallia , P. Gibbs , D. Le , R. L. Giuntoli , M. Goggins , M. D. Hogarty , M. Holdhoff , S. M. Hong , Y. Jiao , H. H. Juhl , J. J. Kim , G. Siravegna , D. A. Laheru , et al., Sci. Transl. Med. 2014, 6, 224ra24. 10.1126/scitranslmed.3007094 PMC4017867 24553385

[21] J. Phallen , M. Sausen , V. Adleff , A. Leal , C. Hruban , J. White , V. Anagnostou , J. Fiksel , S. Cristiano , E. Papp , S. Speir , T. Reinert , M. W. Orntoft , B. D. Woodward , D. Murphy , S. Parpart‐Li , D. Riley , M. Nesselbush , N. Sengamalay , A. Georgiadis , Q. K. Li , M. R. Madsen , F. V. Mortensen , J. Huiskens , C. Punt , N. van Grieken , R. Fijneman , G. Meijer , H. Husain , R. B. Scharpf , et al., Sci. Transl. Med. 2017, 9, eaan2415. 28814544 10.1126/scitranslmed.aan2415 PMC6714979

[22] S. J. Dawson , D. W. Tsui , M. Murtaza , H. Biggs , O. M. Rueda , S. F. Chin , M. J. Dunning , D. Gale , T. Forshew , B. Mahler‐Araujo , S. Rajan , S. Humphray , J. Becq , D. Halsall , M. Wallis , D. Bentley , C. Caldas , N. Rosenfeld , N. Engl. J. Med. 2013,368, 1199. 23484797 10.1056/NEJMoa1213261

[23] E. Giroux Leprieur , G. Herbretau , C. Dumenil , C. Julie , V. Giraud , S. Labrune , J. Dumoulin , J. Tisserand , J. F. Emile , H. Blons , T. Chinet , OncoImmunology 2018, 7, e1424675. 29721388 10.1080/2162402X.2018.1424675 PMC5927532

[24] A. M. Newman , S. V. Bratman , J. To , J. F. Wynne , N. C. Eclov , L. A. Modlin , C. L. Liu , J. W. Neal , H. A. Wakelee , R. E. Merritt , J. B. Shrager , B. W. Loo Jr. , A. A. Alizadeh , M. Diehn , Nat. Med. 2014, 20, 548. 24705333 10.1038/nm.3519 PMC4016134

[25] A. M. Newman , A. F. Lovejoy , D. M. Klass , D. M. Kurtz , J. J. Chabon , F. Scherer , H. Stehr , C. L. Liu , S. V. Bratman , C. Say , L. Zhou , J. N. Carter , R. B. West , G. W. Sledge , J. B. Shrager , B. W. Loo Jr. , J. W. Neal , H. A. Wakelee , M. Diehn , A. A. Alizadeh , Nat. Biotechnol. 2016, 34, 547. 27018799 10.1038/nbt.3520 PMC4907374

[26] C. Abbosh , N. J. Birkbak , G. A. Wilson , M. Jamal‐Hanjani , T. Constantin , R. Salari , J. Le Quesne , D. A. Moore , S. Veeriah , R. Rosenthal , T. Marafioti , E. Kirkizlar , T. B. K. Watkins , N. McGranahan , S. Ward , L. Martinson , J. Riley , F. Fraioli , M. Al Bakir , E. Gronroos , F. Zambrana , R. Endozo , W. L. Bi , F. M. Fennessy , N. Sponer , D. Johnson , J. Laycock , S. Shafi , J. Czyzewska‐Khan , A. Rowan , et al., Nature 2017, 545, 446.28445469

[27] I. Kinde , J. Wu , N. Papadopoulos , K. W. Kinzler , B. Vogelstein , Proc. Natl. Acad. Sci. U. S. A. 2011, 108, 9530. 21586637 10.1073/pnas.1105422108 PMC3111315

[28] A. Stahlberg , P. M. Krzyzanowski , J. B. Jackson , M. Egyud , L. Stein , T. E. Godfrey , Nucleic Acids Res. 2016, 44, e105. 27060140 10.1093/nar/gkw224 PMC4914102

[29] H. Shi , W. Hugo , X. Kong , A. Hong , R. C. Koya , G. Moriceau , T. Chodon , R. Guo , D. B. Johnson , K. B. Dahlman , M. C. Kelley , R. F. Kefford , B. Chmielowski , J. A. Glaspy , J. A. Sosman , N. van Baren , G. V. Long , A. Ribas , R. S. Lo , Cancer Discovery 2014, 4, 80. 24265155 10.1158/2159-8290.CD-13-0642 PMC3936420

[30] E. M. Van Allen , N. Wagle , A. Sucker , D. J. Treacy , C. M. Johannessen , E. M. Goetz , C. S. Place , A. Taylor‐Weiner , S. Whittaker , G. V. Kryukov , E. Hodis , M. Rosenberg , A. McKenna , K. Cibulskis , D. Farlow , L. Zimmer , U. Hillen , R. Gutzmer , S. M. Goldinger , S. Ugurel , H. J. Gogas , F. Egberts , C. Berking , U. Trefzer , C. Loquai , B. Weide , J. C. Hassel , S. B. Gabriel , S. L. Carter , G. Getz , L. A. Garraway , D. Schadendorf , Cancer Discovery 2014, 4, 94. 24265153 10.1158/2159-8290.CD-13-0617 PMC3947264

[31] H. Rizos , A. M. Menzies , G. M. Pupo , M. S. Carlino , C. Fung , J. Hyman , L. E. Haydu , B. Mijatov , T. M. Becker , S. C. Boyd , J. Howle , R. Saw , J. F. Thompson , R. F. Kefford , R. A. Scolyer , G. V. Long , Clin. Cancer Res. 2014, 20, 1965. 24463458 10.1158/1078-0432.CCR-13-3122

[32] D. Mittal , M. M. Gubin , R. D. Schreiber , M. J. Smyth , Curr Opin Immunol. 2014, 27, 16. 24531241 10.1016/j.coi.2014.01.004 PMC4388310

[33] V. Anagnostou , K. N. Smith , P. M. Forde , N. Niknafs , R. Bhattacharya , J. White , T. Zhang , V. Adleff , J. Phallen , N. Wali , C. Hruban , V. B. Guthrie , K. Rodgers , J. Naidoo , H. Kang , W. Sharfman , C. Georgiades , F. Verde , P. Illei , Q. K. Li , E. Gabrielson , M. V. Brock , C. A. Zahnow , S. B. Baylin , R. B. Scharpf , J. R. Brahmer , R. Karchin , D. M. Pardoll , V. E. Velculescu , Cancer Discovery. 2017, 7, 264. 28031159 10.1158/2159-8290.CD-16-0828 PMC5733805

[34] Q. Jia , W. Wu , Y. Wang , P. B. Alexander , C. Sun , Z. Gong , J. N. Cheng , H. Sun , Y. Guan , X. Xia , L. Yang , X. Yi , Y. Y. Wan , H. Wang , J. He , P. A. Futreal , Q. J. Li , B. Zhu , Nat. Commun. 2018, 9, 5361. 30560866 10.1038/s41467-018-07767-w PMC6299138

[35] A. S. Morrissy , F. M. G. Cavalli , M. Remke , V. Ramaswamy , D. J. H. Shih , B. L. Holgado , H. Farooq , L. K. Donovan , L. Garzia , S. Agnihotri , E. N. Kiehna , E. Mercier , C. Mayoh , S. Papillon‐Cavanagh , H. Nikbakht , T. Gayden , J. Torchia , D. Picard , D. M. Merino , M. Vladoiu , B. Luu , X. Wu , C. Daniels , S. Horswell , Y. Y. Thompson , V. Hovestadt , P. A. Northcott , D. T. W. Jones , J. Peacock , X. Wang , et al., Nat. Genet. 2017, 49, 780. 28394352 10.1038/ng.3838 PMC5553617

[36] Z. Hu , P. A. Ott , C. J. Wu , Nat. Rev. Immunol. 2018, 18, 168. 29226910 10.1038/nri.2017.131 PMC6508552

[37] W. Lv , X. Wei , R. Guo , Q. Liu , Y. Zheng , J. Chang , T. Bai , H. Li , J. Zhang , Z. Song , D. S. Cram , D. Liang , L. Wu , Clin. Chem. 2015, 61, 172. 25376582 10.1373/clinchem.2014.229328

[38] S. Chen , Y. Zhou , Y. Chen , J. Gu , Bioinformatics 2018, 34, i884. 30423086 10.1093/bioinformatics/bty560 PMC6129281

[39] A. McKenna , M. Hanna , E. Banks , A. Sivachenko , K. Cibulskis , A. Kernytsky , K. Garimella , D. Altshuler , S. Gabriel , M. Daly , M. A. DePristo , Genome Res. 2010, 20, 1297. 20644199 10.1101/gr.107524.110 PMC2928508

[40] E. Letouze , J. Shinde , V. Renault , G. Couchy , J. F. Blanc , E. Tubacher , Q. Bayard , D. Bacq , V. Meyer , J. Semhoun , P. Bioulac‐Sage , S. Prevot , D. Azoulay , V. Paradis , S. Imbeaud , J. F. Deleuze , J. Zucman‐Rossi , Nat. Commun. 2017, 8, 1315. 29101368 10.1038/s41467-017-01358-x PMC5670220

[41] K. Wang , M. Li , H. Hakonarson , Nucleic Acids Res. 2010, 38, e164. 20601685 10.1093/nar/gkq603 PMC2938201

[42] A. Szolek , B. Schubert , C. Mohr , M. Sturm , M. Feldhahn , O. Kohlbacher , Bioinformatics 2014, 30, 3310. 25143287 10.1093/bioinformatics/btu548 PMC4441069

[43] H. Li , B. Handsaker , A. Wysoker , T. Fennell , J. Ruan , N. Homer , G. Marth , G. Abecasis , R. Durbin , Bioinformatics 2009, 25, 2078. 19505943 10.1093/bioinformatics/btp352 PMC2723002

