# Supplementary material for: Tracking Neoantigens by Personalized Circulating Tumor DNA Sequencing during Checkpoint Blockade Immunotherapy in Non‐Small Cell Lung Cancer
Source: Adv Sci (Weinh). 2020 Mar 26;7(9):1903410. doi: 10.1002/advs.201903410 (PMC7201246; doi:10.1002/advs.201903410)
Supplement: Supplementary file 1 — Supporting Information [file ADVS-7-1903410-s001.pdf]

Copyright WILEY-VCH Verlag GmbH & Co. KGaA, 69469 Weinheim, Germany, 2020.

ADVANCED SCIENCE
Open Access

Supporting Information

for *Adv. Sci.,* DOI: 10.1002/advs.201903410

Tracking Neoantigens by Personalized Circulating Tumor DNA Sequencing during Checkpoint Blockade Immunotherapy in Non-Small Cell Lung Cancer

Qingzhu Jia, Luting Chiu, Shuangxiu Wu, Jian Bai, Lina Peng, Linpeng Zheng, Rui Zang, Xueqin Li, Bibo Yuan, Yixing Gao, Dingyong Wu, Xiaohong Li, Lin Wu, Jianguo Sun, Ji He, Bruce W. S. Robinson, and Bo Zhu**

Supporting Information

Tracking Neoantigens by Personalized Circulating Tumor DNA Sequencing during Checkpoint Blockade Immunotherapy in Non-small Cell Lung Cancer

Qingzhu Jia, Luting Chiu, Shuangxiu Wu, Jian Bai, Lina Peng, Linpeng Zheng, Rui Zang, Xueqin Li, Bibo Yuan, Yixing Gao, Dingyong Wu, Xiaohong Li, Lin Wu, Jianguo Sun, Ji He, Bruce WS Robinson, Bo Zhu**

Qingzhu Jia, Lina Peng, Linpeng Zheng, Rui Zang, Xueqin Li, Bibo Yuan, Yixing Gao, Dingyong Wu, Xiaohong Li, Jianguo Sun, Bo Zhu

Institute of Cancer, Xinqiao Hospital, The Army Medical University, Chongqing 400037, China.

E-mail: sunjg09@aliyun.com; bo.zhu@tmmu.edu.cn

Qingzhu Jia, Lina Peng, Linpeng Zheng, Yixing Gao, Jianguo Sun, Bo Zhu

Chongqing Key Laboratory of Immunotherapy, Chongqing 400037, China.

Luting Chiu, Shuangxiu Wu, Jian Bai

Berry Oncology Corporation. Beijing 100102, China.

Ji He

GeneCast Biotechnology Co., Ltd. , 35 North Haidian Rd, HealthWork Suite 901, Beijing, China

Bruce WS Robinson

University of Western Australia

Bruce WS Robinson

National Centre for Asbestos Related Diseases

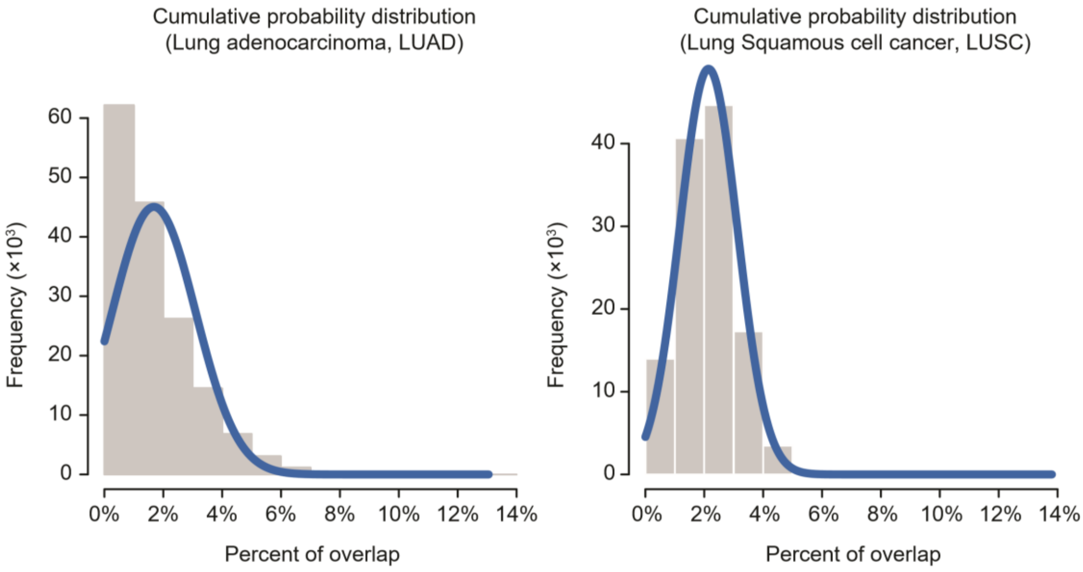

Figure S1. Inter-individual overlap in the mutation spectrum for patients with lung adenocarcinoma (LUAD) or lung squamous cell cancer (LUSC).

Percentage of overlap was calculated as the ratio between numbers of intersected and union genes. Histogram showing the distribution of percentages of overlap for all patients with LUAD (left panel) or LUSC (right panel) in TCGA database. Blue line, fitting curve.

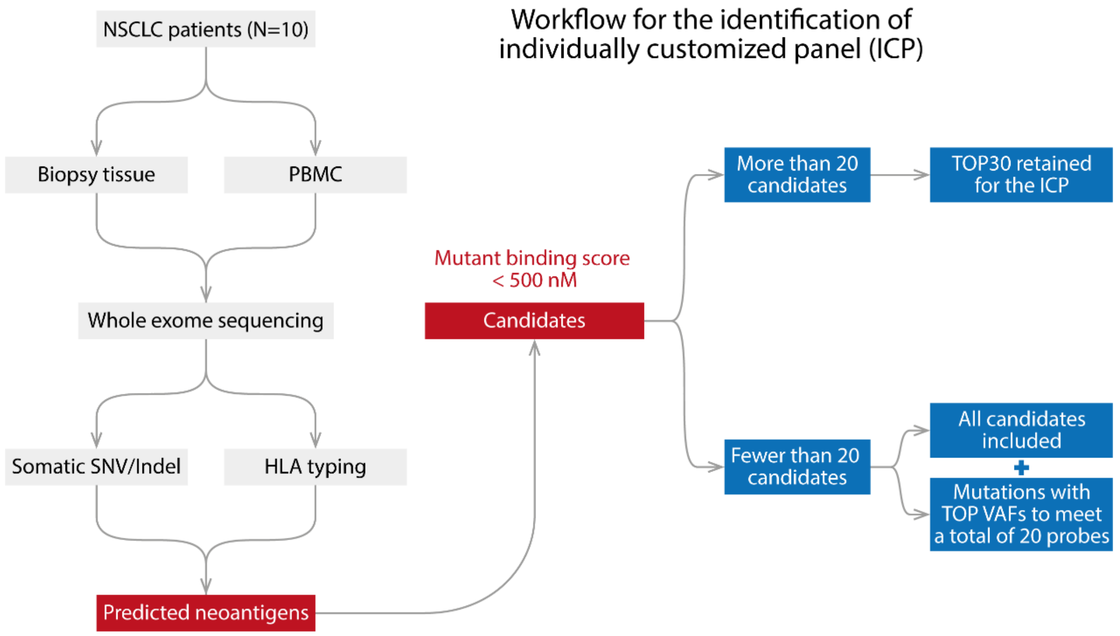

Figure S2. Workflow for the design of individually customized panels (ICP)

Protocol for the design of an ICP for each patient. PBMC, peripheral blood mononuclear cell.

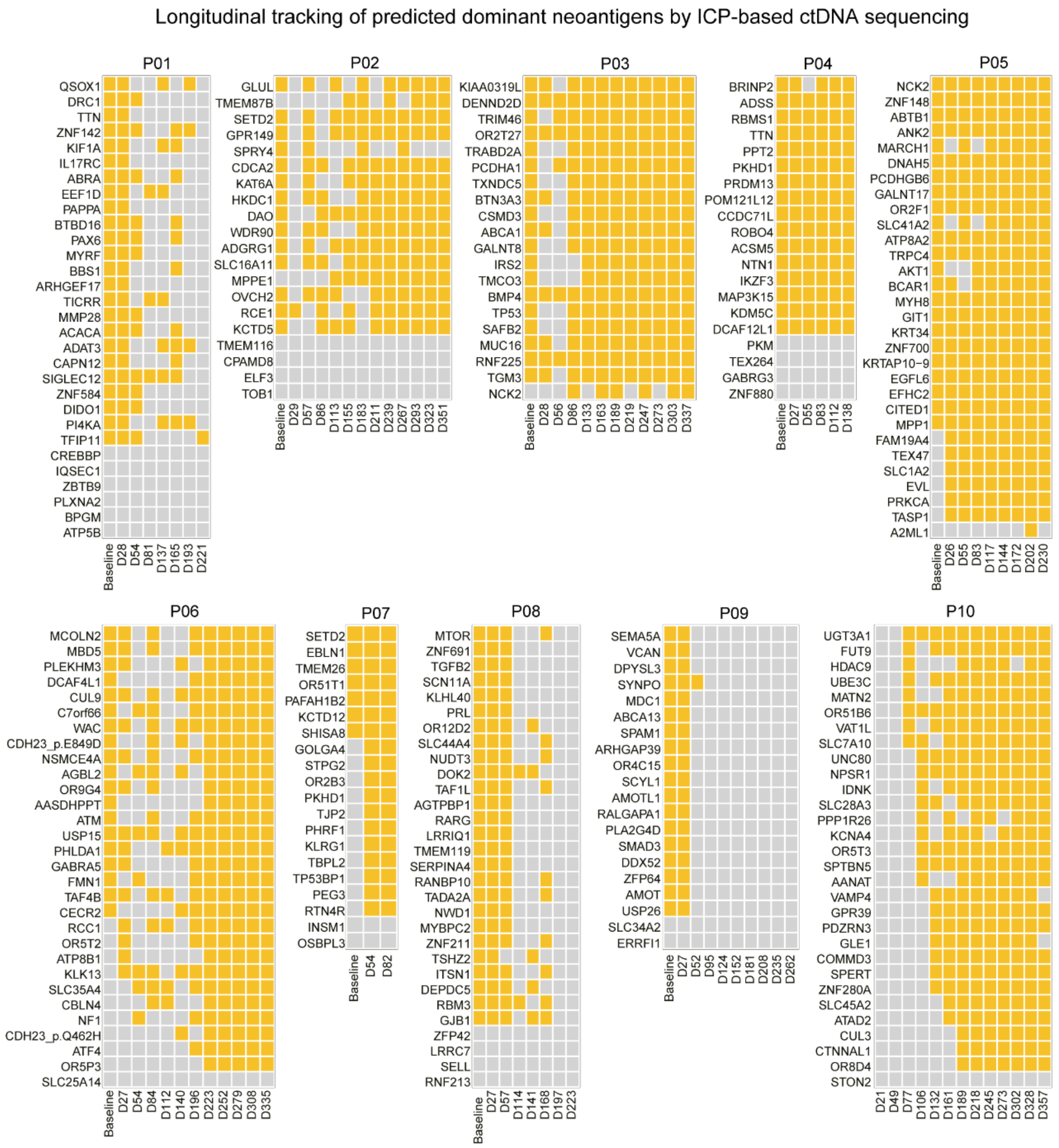

Figure S3. Longitudinal monitoring of circulating predicted dominant neoantigens

Heatmap showing the presence of neoantigens involved in ICP-based ctDNA sequencing during the treatment course. Yellow square, detected; grey square, not detected. Baseline and every sampling time point are labelled.

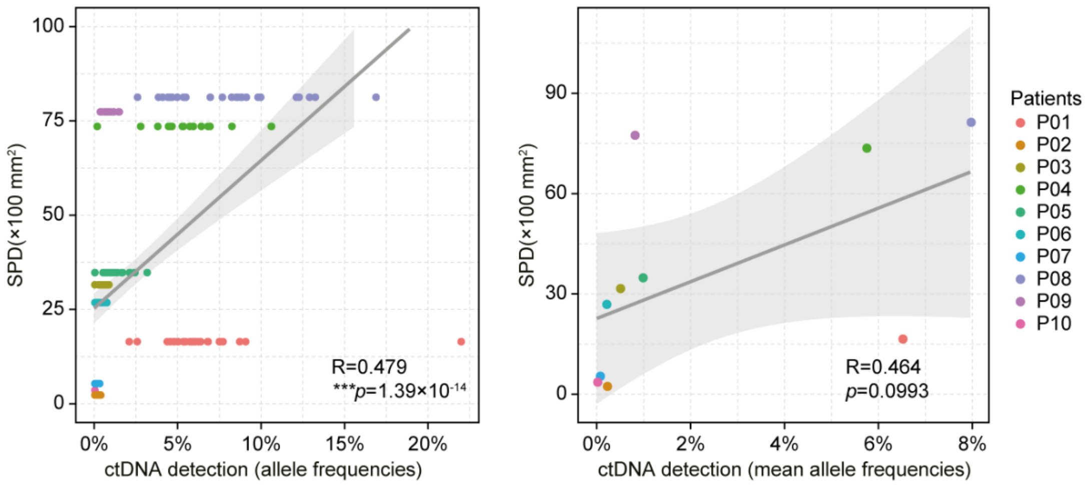

Figure S4. Correlation between ctDNA and tumor burden at baseline.

Scatterplot showing the correlation between the ctDNA concentration and tumor burden. Left panel, each dot represents a detected gene for each patient; right panel, each dot represents the mean MAF for each patient. Tumor burden was measured as the sum of the product of perpendicular diameters. Correlation coefficients and *p*-values are labelled.

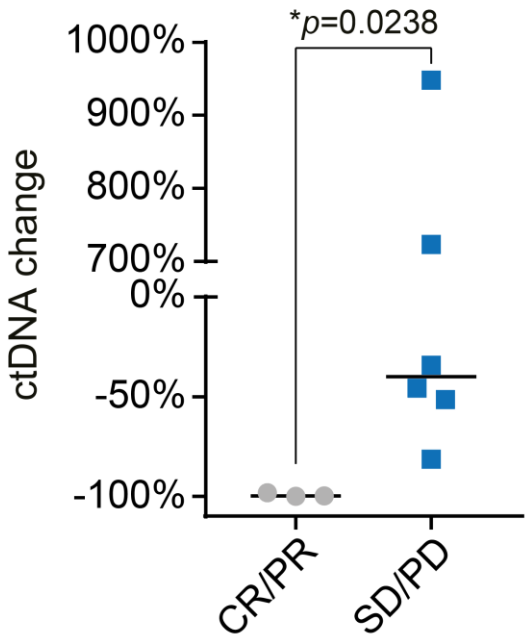

Figure S5. ctDNA change with respect to the objective response.

Plot shows the magnitude of the change in ctDNA against the objective response from baseline levels to 8 weeks after the first ICB administration. Clinical response was evaluated at 8 weeks according to the RECIST 1.1 criteria. P10 was excluded owing to the lack of a baseline plasma sample. CR, complete response; PR, partial response; SD, stable disease; PD, progression of disease. Statistics are based on two-tailed Mann–Whitney *U*-test.

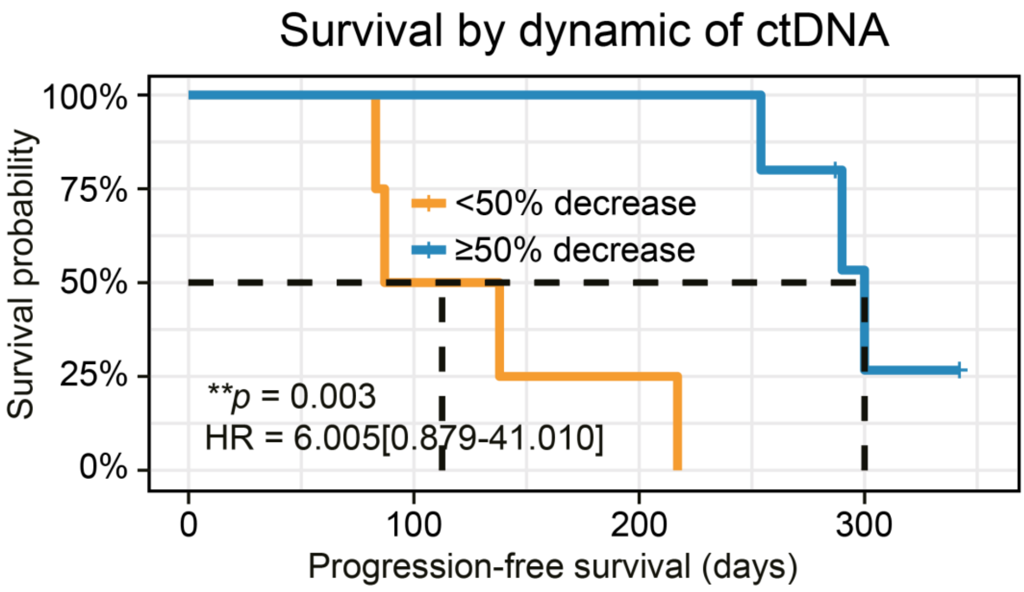

Figure S6. Survival by ctDNA dynamics

Survival analysis with respect to the dynamic change in ctDNA. Patients were grouped by whether or not ctDNA showed a decrease of more than 50% at 8 weeks in comparison to the baseline level. The ctDNA concentration was estimated by the mean MAF for all detected mutations. P10 was excluded owing to the lack of a baseline plasma sample. Kaplan–Meier survival plots, *p*-value from log-rank tests, and hazard ratios with 95% confidence interval (CI) are shown.

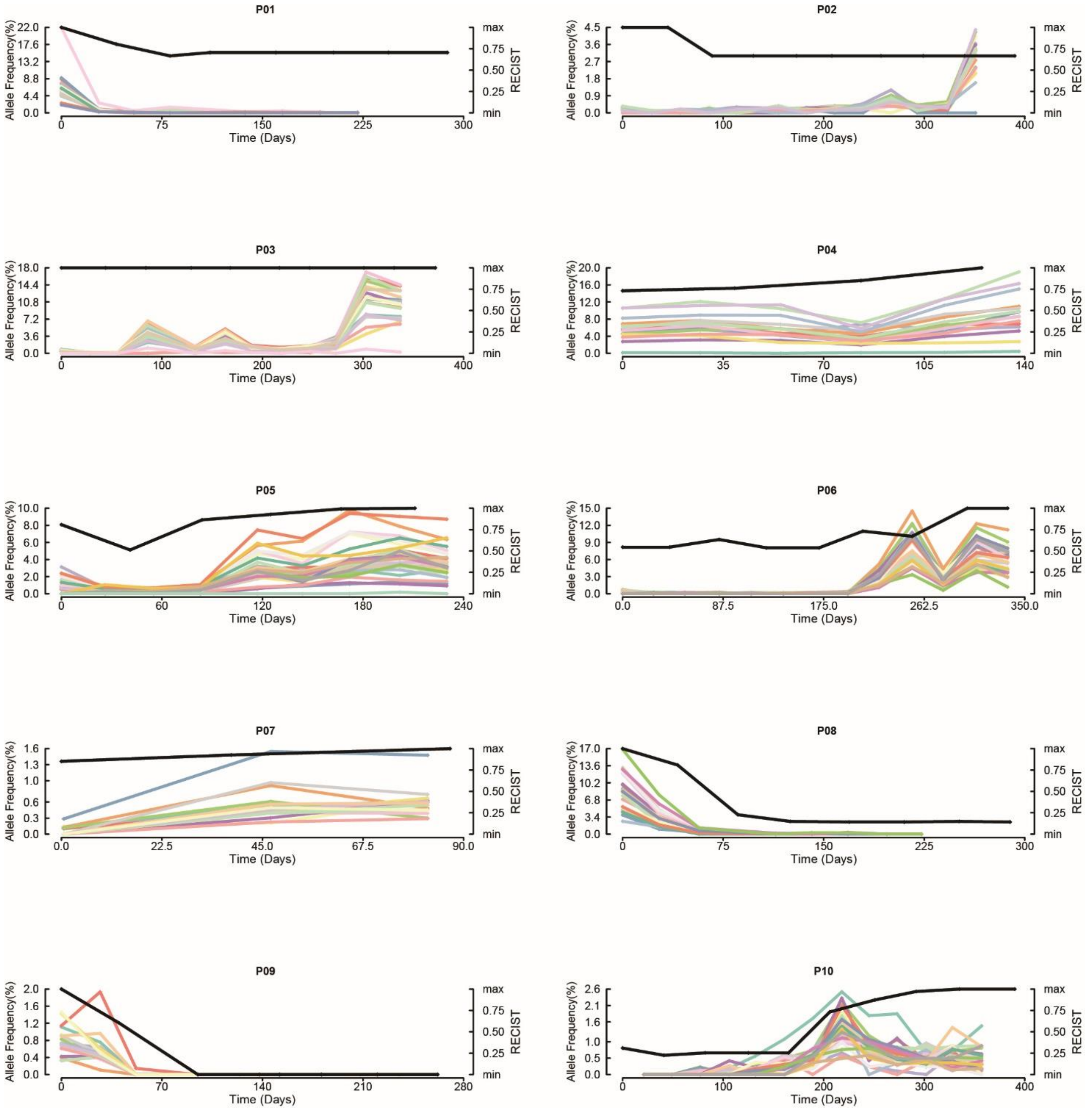

Figure S7. Association between RECIST-measured tumor burden and ctDNA changes during the course of follow-up

Sequential neoantigen ctDNA-assessment and tumor burden during treatment. The dynamic change in the ctDNA allele frequencies are presented as lines of different colors. Tumor burden was quantified as the sum of longest diameter of evaluable lesions according to RECIST criteria, and illustrated as black lines for each patient. RECIST-measured tumor burden were subjected to linear scaling.

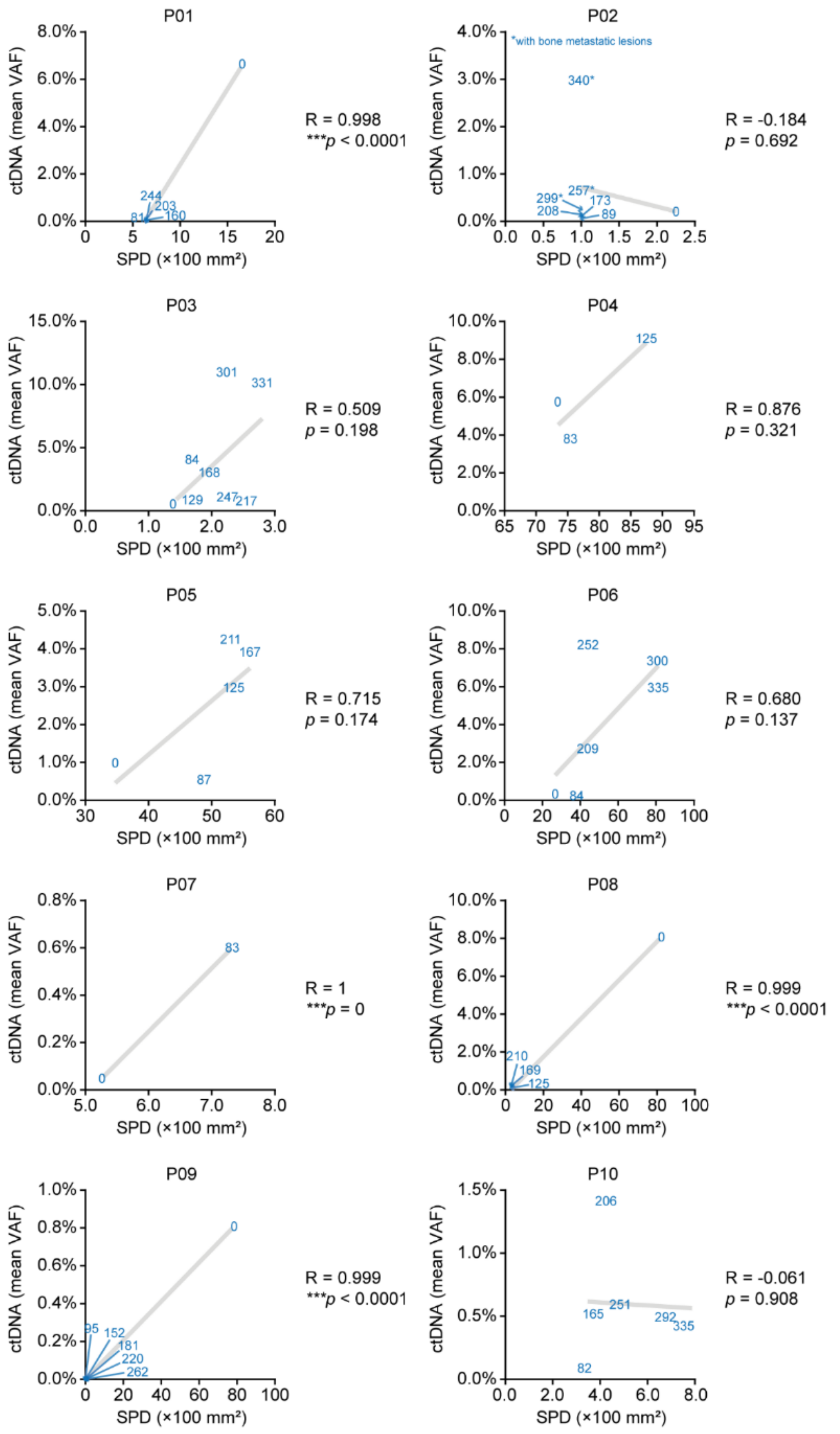

Figure S8. Correlation between tumor burden and ctDNA concentration for individual

Scatter plot displaying tumor burden against the nearest available ctDNA concentration for each patient. Definition of the nearest available ctDNA concentration: no more than ±5 days from the

radiological scan within 100 days; no more than ±10 days from the radiological scan in 101–200 days, and no more than ±20 days from the radiological scan after 200 days from the first infusion of ICB. Each dot is labeled as the time point of the radiological scan; grey lines, fitting lines. *R*-coefficient and *p*-value for Pearson correlation analyses are also presented.

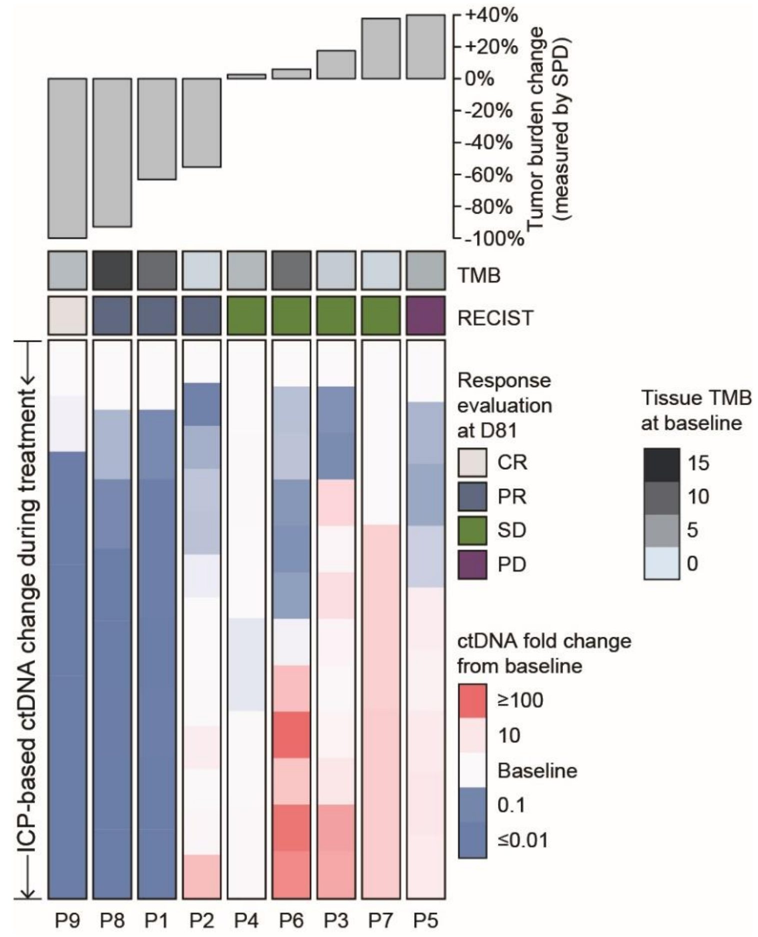

Figure S9. Relevance of the ctDNA change to the tumor burden.

Correlation between the magnitude of the change in SPD-measured tumor burden and ctDNA-assessed neoantigenic evolution after ICB administration. Grey bar plot in **upper panel**, fold change of tumor burden from the baseline to 12 weeks after the first cycle of administration. **Lower panel**, heatmap showing the tendency of ctDNA-assessed neoantigenic evolution from the baseline assay to the last serological follow-up. Each lane represented the ctDNA change for one patients serially. Each color square within the lane represented the ctDNA level in each serological following up. Baseline tissue TMB and best response for each patient are annotated above the heatmap. Color gradient indicates change in tumor ctDNA. All patients are ordered by their tumor shrinkage at the 12-week surveillance scan.

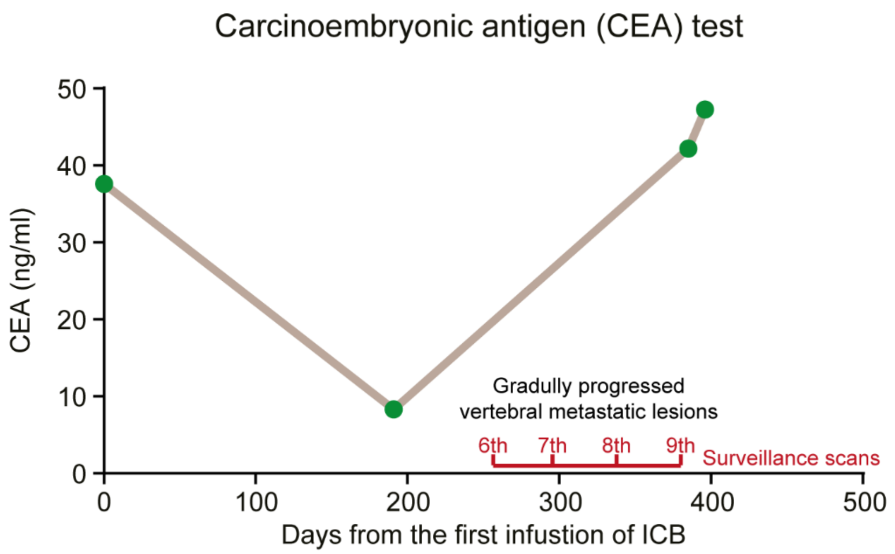

Figure S10. Dynamic change in carcinoembryonic antigen (CEA)

Line and dots show the concentration of CEA for patient P2 during the follow-up period. Red ticks show the timepoints of MRI scans for metastatic vertebral lesions. Reference range of CEA, 0–6 ng ml^{-1}.
